# A novel deep learning model with transformer architectures to enable multi‐scale whole genome sequence analysis for Alzheimer's disease dementia prediction

**DOI:** 10.1002/alz70855_099459

**Published:** 2025-12-23

**Authors:** Eun Hye Lee, Taeho Jo

**Affiliations:** ^1^ Department of Radiology and Imaging Sciences, Indiana University School of Medicine, Indianapolis, IN, USA; ^2^ Indiana Alzheimer Disease Research Center, Indiana University School of Medicine, Indianapolis, IN, USA; ^3^ Department of Radiology and Imaging Sciences, Indiana Alzheimer's Disease Research Center, Center for Neuroimaging, Indiana University School of Medicine, Indianapolis, IN, USA

## Abstract

**Background:**

The importance of early prediction of Alzheimer's disease (AD) is emerging, and a genomic approach provides a promising path to this goal. One limitation is the high dimensionality of genomic data, which remains incompletely understood. Deep learning (DL) models hold potential for processing and interpreting such complex data. This study aimed to develop a novel DL model using a transformer architecture for predicting AD dementia based solely on whole genome sequencing (WGS) data.

**Method:**

We analyzed 1,050 WGS data from the Alzheimer's Disease Neuroimaging Initiative (ADNI) and ADNI‐WGS‐2 with the Alzheimer's Disease Sequencing Project Follow‐Up Study (ADSP‐FUS1‐ADNI‐WGS‐2) (443 cognitively normal; 607 AD dementia). A 1,000,000 base pair segment of WGS data surrounding the APOE gene on chromosome 19 was utilized. Our DL model comprised three steps: (1) Window‐based step: calculation of attention scores using windowed WGS data. (2) Annotation‐enhanced step: analysis of attention scores across multiple layers of selected SNPs, incorporating annotated information to capture distant‐range influences, and generate global attention weights. (3) Prediction performance enhancement using graph convolutional network (GCN): a graph with key SNPs from the previous steps and their relationships was analyzed by GCN to enhance performance. The area under curves (AUCs) for AD dementia prediction from the window‐based step (local relationship patterns) and the entire model (integrating local and distant‐range relationships) were compared. Key SNPs relevant to ADD prediction were identified.

**Results:**

The window‐based step using 40bp‐sized windows yielded limited accuracy (AUC = 0.53), while the complete model showed significant improvement (AUC = 0.68). Among the top 25 SNPs identified via feature importance scores from the complete model, 9 were newly discovered compared to the SNPs from the window‐based step. Key SNPs included rs429358, rs120074114, rs120074111, and rs5122 located in *APOE* or *APOC* genes.

**Conclusion:**

Our DL model enabled multi‐scale analysis of WGS data and improved AD dementia prediction accuracy using WGS data and identified different significant SNPs by integrating local and distant‐range relationships. This study investigated the potential of DL‐based models with transformer architecture in early prediction of AD and established a foundation for efficiently processing WGS data.

